# A Hemophagocytic Lymphohistiocytosis Case with Newly Defined UNC13D (c.175G>C; p.Ala59Pro) Mutation and a Rare Complication

**DOI:** 10.4274/tjh.2014.0416

**Published:** 2015-12-03

**Authors:** Yasemin Işık Balcı, Funda Özgürler Akpınar, Aziz Polat, Fethullah Kenar, Bianca Tesi, Tatiana Greenwood, Nagihan Yalçın, Ali Koçyiğit

**Affiliations:** 1 Pamukkale University Faculty of Medicine, Department of Pediatric Hematology, Denizli, Turkey; 2 Pamukkale University Faculty of Medicine, Department of Pediatrics, Denizli, Turkey; 3 Pamukkale University Faculty of Medicine, Department of Otorhinolaryngology, Denizli, Turkey; 4 Karolinska University Hospital Huddinge, Stockholm, Sweden; 5 Pamukkale University Faculty of Medicine, Department of Pathology, Denizli, Turkey; 6 Pamukkale University Faculty of Medicine, Department of Radiology, Denizli, Turkey

**Keywords:** Hemophagocytic lymphohistiocytosis, Invasive aspergillosis infection, UNC13D (c.175G>C; p.Ala59Pro)

## Abstract

Hemophagocytic lymphohistiocytosis (HLH) represents a severe hyperinflammatory condition with cardinal symptoms of prolonged fever, cytopenias, hepatosplenomegaly, and hemophagocytosis by activated, morphologically benign macrophages with impaired function of natural killer cells and cytotoxic T lymphocytes. A 2-month-old girl, who was admitted with fever, was diagnosed with HLH and her genetic examination revealed a newly defined mutation in the UNC13D (c.175G>C; p.Ala59Pro) gene. She was treated with dexamethasone, etoposide, and intrathecal methotrexate. During the second week of treatment, after three doses of etoposide, it was noticed that there was a necrotic plaque lesion on the soft palate. Pathologic examination of debrided material in PAS and Grocott staining revealed lots of septated hyphae, which was consistent with aspergillosis infection. Etoposide was stopped and amphotericin B treatment was given for six weeks. HLH 2004 protocol was completed to eight weeks with cyclosporine A orally. There was no patient with invasive aspergillosis infection as severe as causing palate and nasal septum perforation during HLH therapy. In immuncompromised patients, fungal infections may cause nasal septum perforation and treatment could be achieved by antifungal therapy and debridement of necrotic tissue.

## Abstract (tr)

Hemofagositik lenfohistiositoz (HLH) uzamış ateş, sitopeni, hepatosplenomegali semptomları ile seyreden, active olmuş, morfolojik olarak benign makrofaj ve doğal öldürücü hücreler ile sitotosik T lenfosit fonksiyon bozukluğu sonucu gelişen hiperenflamatuvar bir durumdur. İki aylık düşmeyen ateş yakınması ile başvuran hasta HLH tanısı aldı ve hastanın genetik incelemesinde UNC13D (c.175G>C; p.Ala59Pro) geninde yeni tanımlanan bir mutasyon saptandı. Hastaya deksamatazon, etopozit ve intratekal metotreksat tedavileri başlandı. Tedavinin 2. haftasında, üç doz etopozit aldıktan sonra, yumuşak damakta plak lezyonu fark edildi ve bu nekrotik lezyon debride edildi. Debridman materyalinin patolojik incelemesinin PAS, Grocott boyamasında aspergilloz enfeksiyonu ile uyumlu olarak çok sayıda septalı hif görüldü. Etopozid tedavisi sonlandırılarak altı hafta boyunca amphotericin B tedavisi verildi. HLH 2004 tedavi protokolü oral siklosporin ile sekiz haftaya tamamlandı. HLH tedavisi sırasında yumuşak damak perforasyonuna neden olacak kadar ağır aspergilloz enfeksiyonu geçiren bir olgu bildirilmemiştir. İmmünyetmezlikli hastada mantar enfeksiyonları nazal septum perforasyonuna neden olabilmektedir ve tedavi nekrotik dokunun debridmanı ve antifungal tedavi ile sağlanabilmektedir.

## INTRODUCTION

Hemophagocytic lymphohistiocytosis (HLH) is a severe life-threatening disease precipitated by secretion of cytokines from morphologically benign macrophages, which ends with uncontrolled hyperinflammation with prolonged fever, cytopenias, hepatosplenomegaly, and hemophagocytosis. Elevation of triglycerides, ferritin, lactate dehydrogenase, and transaminase levels and decreases in fibrinogen levels are characteristic findings [1]. Impaired cytotoxic function of T cells and natural killer cells is a known cause of familial forms of HLH. The HLH-2004 protocol with immunomodulatory and cytotoxic drugs is used for treatment of patients with HLH [2]. Importantly, invasive infections have been reported in up to 56% of children with HLH on chemotherapy, with invasive fungal infections causing 50% of deaths among such cases [3].

Here we report an unusual aspergillosis infection with palate and nasal septum perforation following chemotherapy in a patient with familial HLH with a novel mutation in UNC13D. Notably, the fungal infection in our patient was treated successfully with antifungal therapy and surgical debridement.

## CASE PRESENTATION

A 2-month-old girl, born subsequent to a term gestation with unrelated parents and an unremarkable previous history, was referred to our clinic with unremitting fever since 1 month despite repeated intravenous administrations of antibiotics. There was no history of sibling death in her family. On admission, vital signs were normal except body temperature of 38.7 °C. The patient was pale with petechial rashes on the lower extremities. She displayed hepatomegaly (6 cm below costal margin) and splenomegaly (3 cm below costal margin). Informed consent was obtained.

The patient’s laboratory findings were as follows; hemoglobin: 63 g/L, mean corpuscular volume (MCV): 88.9 fL, total leukocyte count: 2.84x109/L, thrombocyte count: 10x109/L, alanine aminotransferase (ALT): 50 IU/L, aspartate aminotransferase (AST): 62 IU/L, total bilirubin: 0.58 mg/dL, direct bilirubin: 0.7 mg/dL, gamma glutamyl transferase: 328 U/L, albumin: 2.9 g/dL, ferritin: 2000 ng/mL, triglyceride: 617 mg/dL, LDL cholesterol: 11 mg/dL, HDL cholesterol: 7 mg/dL, lactate dehydrogenase: 379 U/L, uric acid: 2.3 mg/dL, fibrinogen: 107 mg/dL. Her renal function tests and electrolytes were normal. Her peripheral smear revealed 4% neutrophils, 90% lymphocytes, and 6% monocytes. Absolute neutrophil count was 0.113x109/L. No hemolysis or blasts were visible in her peripheral blood smear. Her transaminase levels increased on the third day of administration (AST: 280 IU/L, ALT: 265 IU/L). Serological studies for infection with Epstein-Barr virus, parvovirus B19, cytomegalovirus, Toxoplasma gondii, rubella, Leishmania, and hepatitis were all negative. Natural killer cell activity and soluble IL-2 level could not be analyzed. Numerous histiocytes showing hemophagocytosis were observed in the bone marrow aspiration smears.

Conclusively, the patient fulfilled a required 5 out of 6 examined diagnostic criteria for the diagnosis of HLH [1]. Accordingly, the patient was treated with the HLH-2004 protocol with dexamethasone, etoposide, and cyclosporine A. Mutation analyses, identifying a novel homozygous variant in UNC13D (c.175G>C; p.Ala59Pro), confirmed a diagnosis of familial HLH. The variant was not found in the healthy population (1000 Genomes database), and it was predicted as possibly damaging by PolyPhen-2 but as tolerated by sorting tolerant from intolerant (SIFT). The father was a heterozygous carrier of the mutation, while the mother could not be tested.

**Figure 1:**
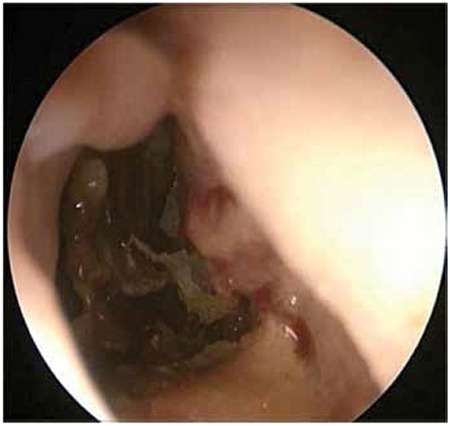
Partial perforation in septal cartilage.

**Figure 2:**
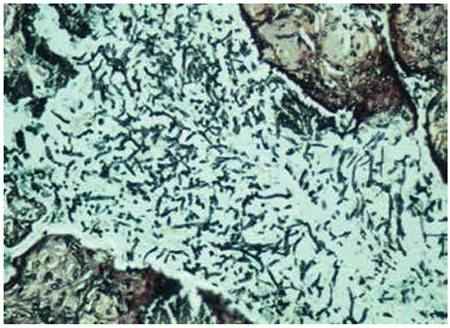
Septated hyphae with 45° angle branching in aspergillosis (Grocott, 100x).

During the second week of treatment, after 3 doses of etoposide at 150 mg/m2/dose, a grossly necrotic soft tissue lesion was noticed on the soft palate and was successfully excised (Figure 1). During the operation an oronasal fistula was revealed, as well as a perforation of the caudal side of the nasal septum along with an abscess formation in the left vestibular floor. Although there was no microbial growth in the necrotic material, microscopic examination of the debrided material in PAS and Grocott staining showed abundant septatedhyphae, consistent with aspergillosis infection (Figure 2). Etoposide was stopped and amphotericin B treatment was given for 6 weeks at a dosage of 3.5 mg/kg/day. The HLH-2004 protocol was followed for 8 weeks with cyclosporine A and dexamethasone orally.

At the most recent follow-up, after 4 months, the patient still presented with a 2-cm hepatosplenomegaly, while her soft palate had successfully epithelialized. However, there is a permanent deformity of her nose. Her laboratory findings were as follows; hemoglobin: 103 g/L, MCV: 77.7 fL, total leukocyte count: 15,280x109/L, thrombocytes: 230,000x109/L, ALT: 32 IU/L, AST: 12 IU/L, ferritin: 916 ng/mL, triglyceride: 421 mg/dL, LDL cholesterol: 78 mg/dL, HDL cholesterol: 20 mg/dL, lactate dehydrogenase: 226 U/L, uric acid: 1.7 mg/dL, fibrinogen: 233 mg/dL. Until bone marrow transplantation she was treated with oral cyclosporine A (at the HLH-2004 protocol dosage), trimethoprim sulfamethoxazole, and fluconazole.

## DISCUSSION AND REVIEW OF THE LITERATURE

Herein we describe the disease course of a patient carrying a novel homozygous UNC13D mutation. Familial HLH cases typically have an earlier presentation, with infectious agents including herpes viruses such as the Epstein-Barr virus precipitating disease. However, in our case, we did not detect an infectious etiological agent. For treatment, chemoimmunotherapy (etoposide, dexamethasone, cyclosporine A, and, for selected patients, intrathecal methotrexate or corticosteroids) is recommended, but for severe disease or familial cases hemopoietic stem cell transplantation is life saving [4].

Opportunistic infections are a common complication of immunosuppression caused by cytotoxic treatment of the disease and by the disease itself. As our case illustrates, HLH patients have a potential risk of developing invasive fungal infections that can be severe. Aspergillus species have emerged as an important cause of life-threatening infections in immunocompromised patients. Highlighting the severity of invasive fungal infections, 6/12 (50%) fatal cases in a study cohort of 18 children with primary HLH were reported to be caused by invasive fungal infections, of which 2 cases were diagnosed with invasive Aspergillus infection first at autopsy [5].

Aspergillus can differentiate into hyphal forms that produce toxins damaging epithelial tissue, leading to invasion of connective and vascular tissue by the fungi, which subsequently can result in thrombosis and ultimately necrosis of hard and soft tissues with perforation. Systemic antifungal therapy and surgical resection or debridement is important for the management of invasive sinonasalaspergillosis. Amphotericin B, voriconazole, and caspofungincan be considered for antifungal therapy [5]. Our case was treated successfully with surgical debridement and 6 weeks of amphotericin B treatment.

In the English literature, the case of a 15-year-old boy who developed fungal infection with nasal septal perforation after bone marrow transplantation for acute myeloid leukemia was reported. He was also treated successfully with surgical debridement and amphotericin B [6].

Our report represents an interesting case of familial HLH caused by a novel homozygous UNC13D mutation and affected by invasive sinonasal aspergillosis.

The UNC13D gene encodes for the Munc13-4 protein, a critical effector of the exocytosis of cytotoxic granules priming cytotoxic granule fusion. Munc13-4 deficiency impairs the delivery of the effector proteins, perforin and granzymes, into the target cells, resulting in defective cellular cytotoxicity and a clinical picture that appears very similar to that of FHL-2 [7]. UNC13D mutations are present in almost 30%-40% of familial HLH cases [8].

To the best of our knowledge, no c.175G>C; p.Ala59Pro mutation in UNC13D has been presented before in the literature. This novel mutation may be responsible for our patient’s severe clinical condition. However, to compare the mutation type and clinical course, there is a need for clinical studies.

We want to emphasize the importance of awareness of the occurrence of potentially life-threatening invasive fungal infections in patients with HLH. Furthermore, this highlights the efficacy of surgical debridement and amphotericin B for successful treatment of fungal infections with focal lesions.
